# Unveiling the Role of Bovine Herpesvirus Type 4 (BHV-4) in Dairy Cow Reproductive Disorders: Insights From a Multifaceted Study in China

**DOI:** 10.1155/tbed/4048149

**Published:** 2024-12-27

**Authors:** Lingling Zhu, Xinjun Dai, Yuxin Lai, Zhigang Guo, Yiping Gu, Jianwei Zhang, Hongyu Wang, Guangjun Chang, Huochun Yao, Zihao Pan

**Affiliations:** ^1^OIE Reference Laboratory for Swine Streptococcosis, College of Veterinary Medicine, Nanjing Agricultural University, Nanjing, China; ^2^College of Veterinary Medicine, Nanjing Agricultural University, Nanjing, China; ^3^Technical Support Center, Modern Farming (Group) Co. Ltd., Anhui, China

**Keywords:** BHV-4, bovine herpesvirus type-4, reproductive disorders, vertical transmission

## Abstract

Bovine herpesvirus type-4 (BHV-4) belongs to the gamma-herpesvirus subfamily, and its association with reproductive disorders in dairy cows is controversial. In this study, 386 whole blood and reproductive swab samples from dairy cows across eight provinces in China were collected. BHV-4 antibody levels in the serum were determined via ELISA, while real-time fluorescent quantitative PCR (qPCR) was employed to detect the DNA of BHV-4, BHV-1, bovine viral diarrhea virus type-I (BVDV-1), and bovine viral diarrhea virus type-II (BVDV-2) in the samples. Additionally, the DNA content of BHV-4 in various tissues of cows and stillborn fetuses was assessed via qPCR. Breeding information for the participating cows was gathered through farmer interviews, and correlation analyses were conducted between the detection results and breeding information. The findings revealed a BHV-4 antibody positive rate of 57.8% and a nucleic acid positive rate of 36.3%. Chi-square analysis revealed a correlation between BHV-4 and BHV-1 infections. Among several pathogens associated with reproductive disorders, only BHV-4 was significantly correlated, whereas BHV-1, BVDV-1, and BVDV-2 were not correlated. Furthermore, the study revealed elevated BHV-4 DNA in the reproductive tracts of cows and stillborn fetuses. Histopathological sections revealed mucosal damage in the vaginal and uterine tissues of stillborn fetuses, a phenomenon not previously reported. In summary, our study provides novel insight into the correlation between BHV-4 and reproductive disorders and presents new evidence that supports the vertical transmission of BHV-4.

## 1. Introduction

Bovine herpesvirus type-4 (BHV-4), a member of the *γ*-herpesvirus subfamily, has garnered significant attention since its initial isolation by Bartha et al. in 1966 [1]. The relationship between BHV-4 and reproductive disorders has been a particularly noteworthy focus of research. Despite this controversy, extensive investigations over the past few decades have provided increasing evidence suggesting a close association between BHV-4 and reproductive disorders in cattle [2–4]. In 2007, Zoltán et al. tested 24 fetal samples from aborted cattle for BHV-4. Their findings revealed that seven out of the 24 samples were positive, suggesting a potential association between BHV-4 and bovine abortion [5]. Furthermore, this association was further supported by over 40 BHV-4 strains isolated from aborted calves by Argentine researchers between 2007 and 2015 [5]. Subsequent research has elucidated the pathogenic mechanisms of BHV-4. A study by Delooz et al. [6, 7] demonstrated the ability of BHV-4 to infect fetuses within the uterus, providing compelling evidence for its association with reproductive disorders. By isolating BHV-4 from the brain of a stillborn calf, Dağalp et al. [8] confirmed the correlation between BHV-4 and reproductive disorders. In addition to its association with miscarriage and stillbirth, BHV-4 has also been found to have a specific tropism for endometrial stromal cells, suggesting that it may be one of the pathogens causing uterine diseases [9, 10]. A study by Klamminger et al. [11] analyzed the prevalence of uterine BHV-4 infection in cows 20–30 days after calving. The results revealed that BHV-4 infection was associated with a reduced chance of insemination and pregnancy in cows as well as an increased risk of clinical endometritis (CE). However, many uncertainties remain regarding the role of BHV-4 in these diseases and how it may contribute to disease.

The first Chinese strain of BHV-4 was not reported until 2019 [12]. Although there is growing evidence linking BHV-4 to reproductive diseases in dairy cows, controversy persists due to its detection in many healthy dairy cows [13]. Therefore, additional evidence is needed to confirm the association of BHV-4 with reproductive diseases in dairy cows, particularly in China, where such reports are limited. Furthermore, most studies on the association of BHV-4 focus solely on this specific virus and overlook other viral factors that pose a threat to the reproductive health of dairy cows, such as BHV-1, bovine viral diarrhea virus type-I (BVDV-1), and bovine viral diarrhea virus type-II (BVDV-2) [14–17]. This oversight may lead to misattribution.

Currently, foundational research on other pathogens associated with bovine reproductive disorders, such as BHV-1 and BVDV, has been progressively reported [18–20]. Numerous vaccines targeting BHV-1 and BVDV have been shown to induce stronger immune responses [21–23]. However, basic research and vaccine development for BHV-4 are still in its early stages, possibly due to incomplete foundational research on BHV-4 and a limited understanding of its pathogenesis and transmission mechanisms. Given the relatively complex pathogenesis and transmission modes of BHV-4, there is a need for in-depth exploration and further research to provide theoretical guidance for the development of BHV-4 vaccines.

Therefore, the objectives of this study were twofold: (i) to determine the correlation between coinfection with various viral pathogens causing reproductive disorders and the relationship between BHV-4 and reproductive disorders in China and (ii) to investigate the transmission mode of BHV-4, confirm its potential for vertical transmission, and identify the tissue tropism of BHV-4 in adult and fetal cows.

## 2. Materials and Methods

### 2.1. Research Subjects

The research was conducted in November 2023 across eight farms located in eight different provinces of China, designated Farm A to Farm H. A Chinese dairy group owns these farms and employs intensive farming systems with a primary focus on the breeding of Holstein–Friesian cattle. All eight farms used artificial insemination for mating and breeding, with semen sourced from a single provider. The eight farms implemented immunization programs for foot-and-mouth disease, bovine ephemeral fever, lumpy skin disease, brucellosis, bovine tuberculosis, bovine *Clostridium perfringens*, and infectious bovine rhinotracheitis (IBR)/bovine viral diarrhea (BVD). For IBR/BVD immunization, the initial vaccination was administered in the third month, followed by a booster in the fourth month. Additionally, immunization was reinforced once 30 days before breeding, and pregnant cattle received immunization 60 days before calving. Importantly, none of the eight farms carried out immunizations against BHV-4.

To explore the correlation between BHV-4 and the breeding state of dairy cows, this study focused on two main subjects: normally raised calves and cows. In this context, a calf is defined as a heifer ~12 months old that has not yet been conceived, whereas a cow is defined as one that is ~15 days postpartum. These herds are normally raised dairy cattle.

### 2.2. Sample and Data Acquisition

Using stratified random sampling, we selected a total of 386 cattle, including 162 calves and 224 cows. Whole blood samples and reproductive tract swabs were collected from each selected animal for further analysis. Blood samples were collected via tail venipuncture (Junnuo, Shandong, China) via disposable syringes (Junnuo, Shandong, China) and transferred into disposable vacuum blood collection tubes containing heparin sodium anticoagulant (Junnuo, Shandong, China). The anticoagulated blood was promptly sent to the laboratory within 24 h and stored at 4°C. Reproductive tract swab samples were collected via flocked swabs (Aidete, Hunan, China) and placed into sampling tubes containing preservation solution (CWBIO, Jiangsu, China). The number of cattle and time of collection were clearly indicated on the sample tube label before sampling. Following animal restraint, the vulva was disinfected before a flocked swab was gently collected and deeply inserted along the genital tract wall. The swab was left in place for 30 s before being slowly rotated and withdrawn. Immediately upon removal, the swab was placed into a sample tube containing a preservation solution. The swab was then broken off at a designated breakpoint, the tube cap was secured, and the sample was sent to the laboratory within 24 h while it was stored at 4°C. The study collected reproductive information from 98 cows at 15 days postpartum, including parity, age in months, average daily milk production, calf birth weight, and the incidence of reproductive disorders. Reproductive disorders primarily include metritis, retained placenta, and birth canal strain (Supporting Information 2).

### 2.3. Serological Analysis, DNA Extraction, and Real-Time Fluorescence Quantitative PCR (qPCR) Analysis

The anticoagulated blood samples were centrifuged at 3000 rpm for 10 min to separate the serum, which was subsequently utilized for ELISA detection. Serum antibody concentrations were quantified with a BHV-4 antibody test kit sourced from ID.vet, France, in strict accordance with the manufacturer's protocol. Experimental validity was confirmed by ensuring that the optical density (OD) value of the positive control exceeded 0.350, and that the ratio of the OD values of the positive and negative controls exceeded 3. The interpretation of sample results as positive or negative was conducted following the provided instructions.

Whole anticoagulated blood samples were combined at a 1 : 1 ratio with reproductive tract swab samples for subsequent DNA extraction. The extraction process was carried out via a fully automated nucleic acid extractor (Vazyme, Nanjing, China). Real-time fluorescent qPCR was conducted via dual-fluorescence qPCR kits (Lijian, Qingdao, China) for bovine herpesvirus-1 (BHV-1) and BHV-4, as well as BVDV-1 and BVDV-2. A QuantStudio 6 fluorescence qPCR instrument (ABI, USA) was utilized for qPCR amplification and detection. The protocol was performed in strict accordance with the provided instructions. Analysis was conducted in the FAM/VIC signal channel. A sample was considered positive for nucleic acid if it exhibited a distinct amplification curve and a Ct value of less than 38. Conversely, the absence of a Ct value indicated a negative result. For samples with Ct values ranging from 38 to 40 and exhibiting a specific amplification curve, initial suspicion was raised. Retesting was then carried out, and if the Ct value remained below 40 with the appearance of a specific amplification curve, the sample was deemed positive for nucleic acid.

### 2.4. BHV-4 Detection in Tissues of BHV-4-Positive Cows and Their Stillborn Fetuses

To ascertain evidence of vertical transmission of BHV-4 and delineate its distribution within adult and fetal bovine tissues, we collected tissues from an adult cow that tested positive for BHV-4 and from an aborted fetal cow, and qPCR analysis was performed on these samples. The tissue samples from BHV-4-positive adult cows included the heart, liver, spleen, lung, kidney, lymphoid tissue, blood, uterine horn, cervix uteri, uterine body, vulva, and vaginal wall. The samples from stillborn fetuses included the heart, liver, lung, kidney, pancreas, trachea, muscle, pericardium, umbilical cord, medulla oblongata, brain, rumen, abomasum, duodenum, jejunum, ileum, cecum, colon, breast, vulva, cervix uteri, and uterine body. Specifically, tissues weighing 0.1 g were soaked in 1 mL of PBS and then transferred into glass homogenate tubes. Tissue homogenization was performed using a homogenizer (MP, USA). An automatic nucleic acid extractor (Vazyme, Nanjing, China) was used to extract DNA from the tissue homogenate. BHV-4 was detected as described in the previous section.

### 2.5. Histopathological Sections of Stillborn Fetuses

Vaginal and uterine tissues from stillborn fetuses were collected at ~1 cm × 1 cm in size and fixed in 4% paraformaldehyde. The samples were dehydrated in 80%, 90%, 95%, and 100% ethanol for 2 h each. The samples were subsequently embedded, sliced, and coated with paraffin before being stained with hematoxylin–eosin. Finally, the sections were observed under a microscope (Zeiss, Germany).

### 2.6. Statistical Analyses

Statistical analyses were conducted via GraphPad Prism 8 and IBM SPSS Statistics 26 software. Normality tests were employed to assess the distributional normality of continuous data, facilitating the selection of an appropriate analytical model. The differences in antibody positivity rates and nucleic acid positivity rates between calves and cows were assessed via independent sample *t*-tests. Chi-square tests were used to investigate the associations between various pathogens, as well as the correlations between reproductive disorders and different pathogens. Pearson's chi-square test was applied when the sample size (*N*) was ≥ 40 and the theoretical frequency (*T*) was ≥ 5. Yates's correction for continuity was utilized when *N* ≥ 40 and 1≤ *T* ≤5. Fisher's exact test was employed when *N* < 40 or *T* < 1.

## 3. Results

### 3.1. Prevalence of BHV-4 in China

**Figure 1:**
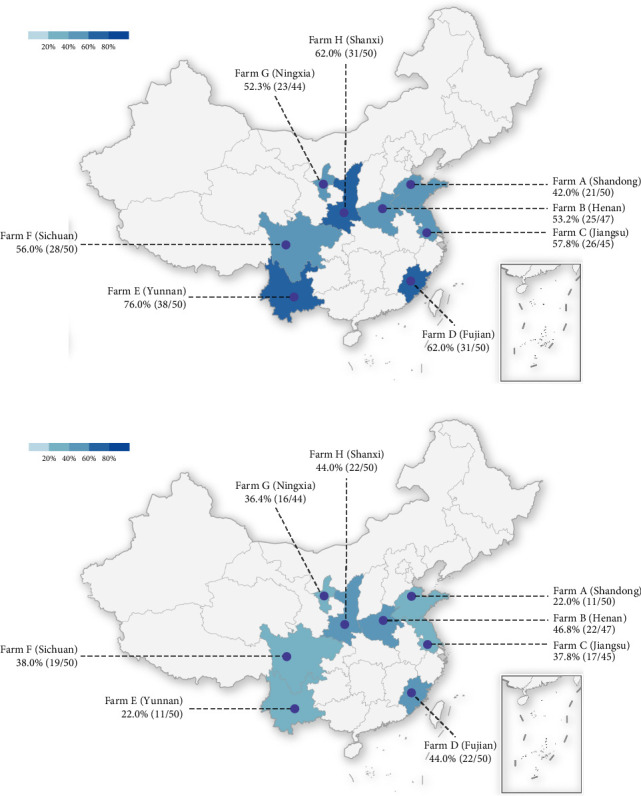
Prevalence of BHV-4: (A) distribution of the positive rate of BHV-4 antibody; (B) distribution of the positive rate of BHV-4 nucleic acid.

This study was conducted to assess the prevalence of BHV-4 in dairy cows across eight provinces in China. A total of 386 whole blood samples and corresponding reproductive tract swabs were collected for serological and nucleic acid testing. Serological testing revealed an overall seropositive rate of 57.8% across the eight provinces, with the highest positivity rate observed in ranches from Yunnan Province at 76%. Additionally, antibody positivity rates exceeded 50% in seven provinces, with three provinces surpassing a 60% positivity rate (Figure 1A). The overall percentage of positive nucleic acid samples across the eight provinces was 36.3%, with Henan, Fujian, and Shanxi exhibiting nucleic acid positivity rates exceeding 40% (Figure 1B). In conclusion, it can be inferred that the prevalence of BHV-4 antibodies is greater than that of BHV-4 nucleic acids in China.

### 3.2. Distribution of BHV-4 in Calves and Cows

**Figure 2:**
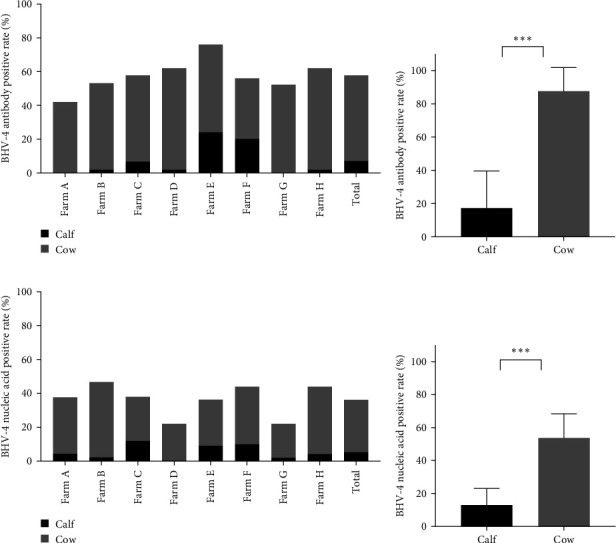
Distribution of BHV-4 in calves and cows. (A) Positive rate of BHV-4 antibody in calves and cows from different farms. (B) Total positive rate of BHV-4 antibody in calves and cows. (C) Positive rate of BHV-4 nucleic acid in calves and cows from different farms. (D) Total positive rate of BHV-4 nucleic acid in calves and cows. *⁣*^*∗∗∗*^*p* < 0.001.

In this study, calves were defined as heifers ~12 months old that had not yet been conceived, whereas cows were considered to be cows ~15 days postpartum. To investigate the prevalence of BHV-4 in calves and cows, a total of 162 whole blood samples and reproductive tract swabs from calves, as well as 224 blood samples and reproductive tract swabs from cows, were collected through stratified random sampling. The results revealed that the antibody-positive rates and nucleic acid-positive rates among cows were higher than those reported for calves across farms located in eight different provinces in China (Figure 2A,C). Specifically, the antibody-positive rate among calves was 17.3%, with a nucleic acid-positive rate of 13.0%. In contrast, the antibody-positive rate among cows was significantly greater at 87.1%, with a nucleic acid-positive rate of 53.1%. Statistical analysis confirmed that there was a significant difference in the positive rates of BHV-4 antibodies and nucleic acids between calves and cows (*p* < 0.001), indicating that these rates were notably higher among cows than among calves (Figure 2B,D). Detailed data are provided in Supporting Information 1.

### 3.3. Relationship Between the Rate of BHV-4 Positivity and Parity

**Figure 3:**
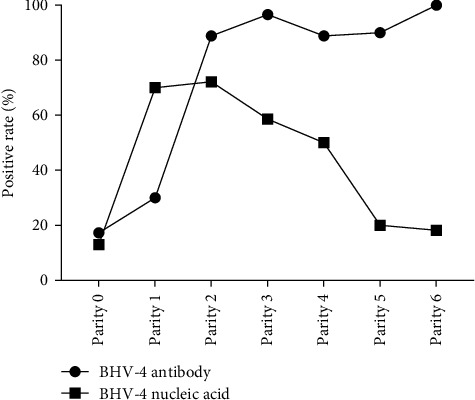
The curve of the antibody-positive rate and nucleic acid-positive rate of BHV-4 at each parity.

To investigate the prevalence of BHV-4 among cattle of varying parity, the cattle were meticulously categorized on the basis of their parity: 0 parity (calves) (*N* = 162), 1 parity (*N* = 10), 2 parity (*N* = 18), 3 parity (*N* = 29), 4 parity (*N* = 18), 5 parity (*N* = 10), and 6 parity (*N* = 11) (Supporting Information 2). Owing to the limited number of samples collected in this study, 7 or 8 parity samples were excluded from the analysis. The positive rates of BHV-4 antibody and nucleic acid in each parity group were calculated, and a parity-BHV-4 positive rate curve was plotted (Figure 3). The positive rate of BHV-4 nucleic acid was observed to significantly increase from 0 parity to 1 parity, reaching its peak at the second parity and then showing a downward trend. The positive rate of BHV-4 antibody increased steadily during the first two parties and then remained relatively stable, suggesting that BHV-4 infection may occur at first parity. Furthermore, when antibody levels were high, the detection rate of BHV-4 nucleic acid began to decrease, indicating that the antibodies produced in response to BHV-4 may have the ability to neutralize virus particles. Additionally, the study revealed that reproductive disorders occurred in cows of all parities (Supporting Information 2), indicating that these diseases are not influenced by parity.

### 3.4. Distribution of Other Pathogens Causing Reproductive Disorders in Cattle

**Figure 4:**
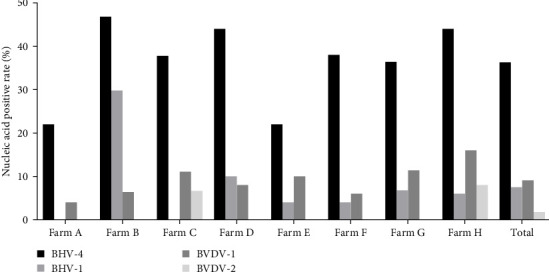
Nucleic acid positivity rates of BHV-4, BHV-1, BVDV-1, and BVDV-2 at each farm.

**Figure 5:**
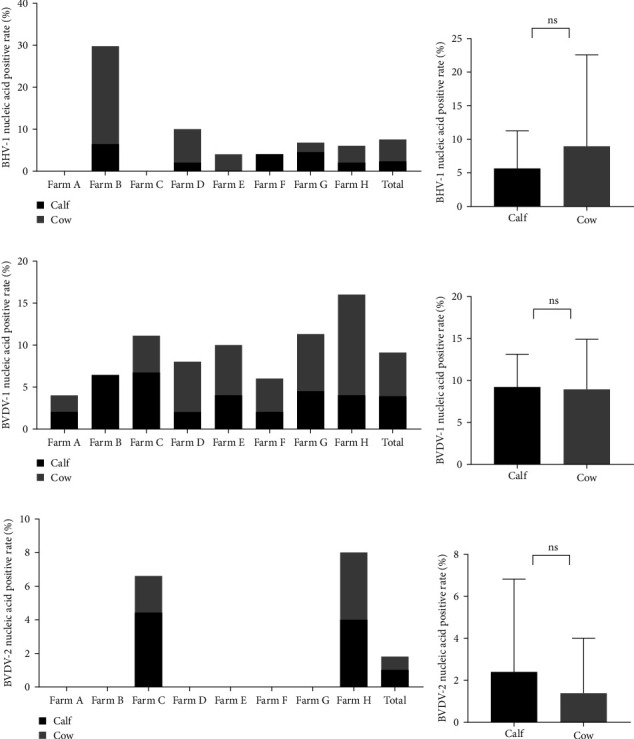
Prevalence of BHV-1, BVDV-1, and BVDV-2 in calves and cows. (A) Positive rate of BHV-1 nucleic acid in calves and cows from different farms. (B) Total positive rate of BHV-1 nucleic acid in calves and cows. (C) Positive rate of BVDV-1 nucleic acid in calves and cows from different farms. (D) Total positive rate of BVDV-1 nucleic acid in calves and cows. (E) Positive rate of BVDV-2 nucleic acid in calves and cows from different farms. (F) Total positive rate of BVDV-2 nucleic acid in calves and cows. ns, no significance.

The samples were tested for viral nucleic acids associated with bovine reproductive disorders, including BHV-1, BVDV-1, and BVDV-2. The results revealed positive rates of 7.5% for BHV-1, 9.1% for BVDV-1, and 1.8% for BVDV-2. The prevalence of BHV-4 was greater than that of BHV-1, BVDV-1, and BVDV-2 across all eight farms (Figure 4). Furthermore, the distributions of BHV-1, BVDV-1, and BVDV-2 did not show a consistent pattern between calves and cows within each farm (Figure 5A,C,E), nor was there a significant difference in their distribution between these two groups (Figure 5B,D,F). Detailed data are provided in Supporting Information 1.

### 3.5. Analysis of the Correlations Between BHV-4 and BHV-1, BVDV-1, and BVDV-2

To investigate the potential relationships between BHV-4 infection and BHV-1, BVDV-1, and BVDV-2, the cattle were categorized into calves and cows for chi-square tests. The results (Table 1) revealed no associations between BHV-4 infection and BVDV-1 (*p*=0.91) or BVDV-2 (*p*=0.41) infection. However, a significant association was found between BHV-4 infection and BHV-1 infection (*p*=0.003), particularly in calves (*p* ≤ 0.001) but not in cows (*p*=0.265). Detailed data are provided in Supporting Information 1.

### 3.6. Correlation Analysis of BHV-4, BHV-1, BVDV-1, and BVDV-2 With Reproductive Disorders and Productivity

Reproductive disorder information was collected from 98 out of 224 individual cows. The associations of BHV-4 antibody and BHV-4 nucleic acid with reproductive disorders were assessed via the chi-square test. The results (Table 2) revealed a significant correlation between positive BHV-4 nucleic acid and the occurrence of reproductive disorders (*p*=0.042), whereas positive BHV-4 antibodies did not correlate with the occurrence of reproductive disorders (*p*=0.830). Furthermore, this study also analyzed the correlations between BHV-1, BVDV-1, and BVDV-2 and reproductive disorders. The results revealed no associations between BHV-1, BVDV-1, or BVDV-2 and reproductive disorders (Table 3). Detailed data are provided in Supporting Information 1.

**Figure 6:**
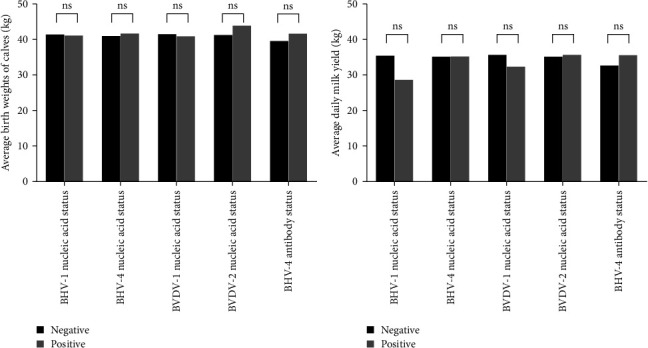
(A, B) The impact of BHV-1, BHV-4, BVDV-1, and BVDV-2 on the production performance of dairy cows. ns, no significance.

In this study, the assessment of production performance included evaluating the average calf birth weight and the average daily milk yield. A total of 78 valid datasets were collected from 224 adult cows, with 75 testing negative for BHV-1 nucleic acid and three testing positive. Additionally, 33 tested negative for BHV-4 nucleic acid, whereas 45 tested positive. Furthermore, there were 67 instances of negative BVDV-1 nucleic acid results and 11 instances of positive BVDV-1 nucleic acid results. Similarly, 76 patients were negative for BVDV-2 nucleic acid, whereas two patients were positive for BVDV-2 nucleic acid. Moreover, nine patients tested negative for BHV-4 antibodies, whereas 69 tested positive. Statistical analysis revealed that BHV-1, BHV-4, BVDV-1, and BVDV-2 did not significantly affect either the average calf birth weight or the average daily milk yield in dairy cows (Figure 6A,B). Detailed data are provided in Supporting Information 2.

### 3.7. Vertical Transmission is a Route of BHV-4 Transmission

The CT values of BHV-4 in various stillborn fetal tissues were determined via real-time fluorescent qPCR (Table 4). BHV-4 nucleic acid was present in all tissues, with blood and lymphoid tissue showing lower CT values than the heart, liver, spleen, lung, and kidney. Additionally, low CT values for BHV-4 were found in the cervix, uterine body, vulva, and vaginal wall, suggesting a greater likelihood of BHV-4 colonization in the reproductive tract. However, notably, higher CT values were detected in the uterine horns.

In stillborn fetuses, BHV-4 nucleic acid was not detected in the liver, lung, pancreas, trachea, muscle, pericardium, bulbar, or jejunum (Table 5). Certain levels of BHV-4 nucleic acid were detected in the brain, heart, kidney, and digestive system, with the exception of the jejunum. Notably, low CT values were detected in the cervix uteri and uterine body of stillborn fetuses, as well as in the umbilical cord tissue. These findings suggest that BHV-4 in cows is likely transmitted to fetal cattle through umbilical cord blood.

**Figure 7:**
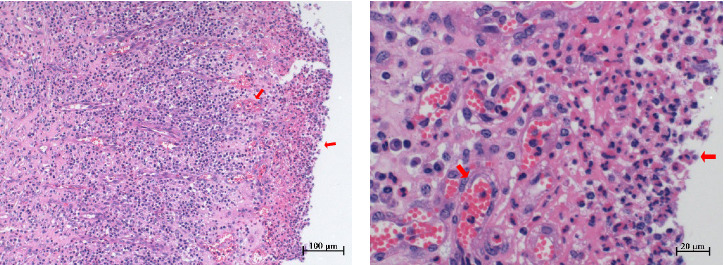
Vaginal histopathological section of a stillborn fetus. Mucosal necrosis of the vaginal tissue, vascular congestion, and infiltration of a significant number of inflammatory cells, including neutrophils, plasma cells, macrophages, and lymphocytes, can occur. (A) Scale, 100 μm. (B) Scale, 20 μm.

**Figure 8:**
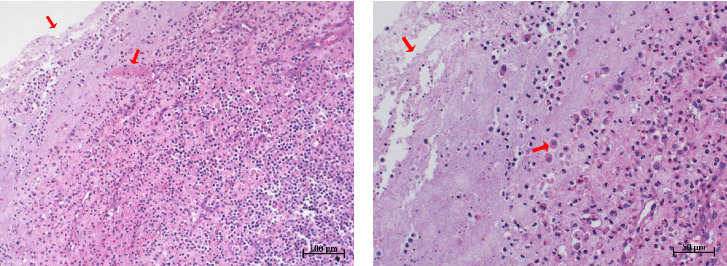
Histopathological section of a stillborn fetal uterus. The sections showed necrosis of the uterine mucosa and infiltration of a large number of inflammatory cells, such as neural cells, plasma cells, macrophages, and lymphocytes. Some macrophages formed vacuoles, and red, round protein substances were present in the cytoplasm. (A) Scale, 100 μm. (B) Scale, 50 μm.

Histopathological sections revealed mucosal necrosis and inflammatory cell infiltration in the vaginal and uterine tissues of stillborn fetuses (Figure 7). Furthermore, vacuolated macrophages with red, round protein substances in the cytoplasm were observed in the uterine tissue sections (Figure 8). These findings suggest that BHV-4 may be transmitted vertically to fetal bovines, resulting in damage to the vagina and uterus.

## 4. Discussion

The potential effects of BHV-4 on dairy cows have yet to be extensively studied. In this context, we conducted the first large-scale study of BHV-4 prevalence across eight ranches owned by a well-known group in China. Our study revealed a high BHV-4 antibody-positive rate of up to 57.8% in the collected samples. These findings are consistent with a 2020 report on BHV-4 prevalence in dairy cows in China, which also revealed a high BHV-4 antibody prevalence in Inner Mongolia (64%) [12]. These results suggest that more than half of all dairy cows in China have been exposed to BHV-4. In contrast, the prevalence of BHV-4 antibodies reported in other countries over the past decade has generally been lower. In 2014, researchers in New Zealand identified 23 BHV-4-positive samples out of 92 serum samples from cows with metritis, resulting in a positive rate of 25% [24].

Similarly, in 2015, Graham et al. [25] reported an antibody-positive rate of 33.3% in Northern Ireland. Reports from Canada, Turkey, and Pakistan indicated positive rates of BHV-4 antibodies of 7.9% [2], 4.16% [26], and 8% [27], respectively, which are lower than those reported in China, highlighting the need for increased attention to the prevention and control of BHV-4 in China. Studies concerning BHV-4 nucleic acid detection are scarce worldwide. In 2014, researchers from New Zealand reported the absence of BHV-4 DNA in peripheral blood mononuclear cells from cows with metritis [24]. A 2022 study conducted in Turkey also failed to detect BHV-4 nucleic acid in aborted and infertile cattle [28]. However, in the same year, Turkish scholars detected 42 positive BHV-4 nucleic acid samples out of 100 white blood cell samples from cows diagnosed with endometritis, resulting in a positive rate of 42% [29]. Our study revealed a positive nucleic acid rate of 36.3% from the collected samples. Variations in antibody and nucleic acid positive rates could be attributed to differences in sampling methods, sample types, and regional distributions.

Additionally, factors such as BHV-4 vaccine coverage, variations in diagnostic techniques, and differences in herd management and sanitation conditions may be significant contributors to these differences. Given the limited sample size and geographical focus of this epidemiological survey, defining the specific prevalence of BHV-4 in China is challenging. BHV-4 may be prevalent in China, and additional epidemiological data are imperative for accurately ascertaining its prevalence in China. The rates of nucleic acid positivity and antibody positivity detected in this study were not consistent and showed considerable variation among cows of different parities. However, this phenomenon aligns with the typical progression of viral infections. During the early stages of infection, the virus rapidly replicates within cells but does not produce sufficient specific antibodies, which makes it difficult to detect antibodies through serological methods at this stage. As infection progresses, antibody levels in the body gradually increase, and immune memory is established. With the activation of the immune system, the levels of viral nucleic acids in the body begin to decrease. Consequently, the positivity rates for BHV-4 nucleic acids and antibodies may not be entirely consistent and can even differ significantly.

To investigate the association of BHV-4 with the reproductive status of cows, we divided the herd into calves and cows. The calves were typically ~12 months of age before conception, whereas the cows were defined as being ~15 days postpartum. Our findings revealed that both the positive rates of BHV-4 antibody and nucleic acid in calves were significantly lower than those in cows. An increase in the antibody-positive rate was observed in cows with parity 2, whereas an increase in the nucleic acid-positive rate was evident as early as parity 1, suggesting that the spread of BHV-4 is rapid and efficient, potentially occurring at first parity, which aligns with previous research from Spanish scientists [30]. Additionally, our study revealed that an elevated level of BHV-4 antibody was associated with a decreased nucleic acid detection rate of BHV-4. These findings indicate that BHV-4 is capable of stimulating the production of neutralizing antibodies, indicating the potential for the development of BHV-4 vaccines. This study provides an approximate estimation of the timing of BHV-4 infection, suggesting that infection likely occurs around the time of first parity. However, since our study did not involve dynamic detection, we were unable to determine the exact time of infection. Therefore, it is crucial to implement dynamic monitoring for BHV-4. Considering that all eight farms utilized consistent fertilization methods and semen sources, we speculated that this phenomenon may be related to bull semen containing BHV-4 pathogens. Alternatively, this issue might also stem from the practices of farm technicians. Technicians might not be adequately sterilized during artificial insemination or pregnancy examinations, leading to widespread transmission of BHV-4. Consequently, dairy farms should prioritize semen quality and ensure proper procedures by technical personnel. Regular testing of semen sources for BHV-4 is essential, and strict disinfection protocols should be followed during artificial insemination and prenatal examinations to minimize the risk of BHV-4 transmission.

Here, we observed a correlation between BHV-4 and BHV-1 infection, particularly in calves. A study conducted in 2021 revealed that the incidence of coinfection with BHV-1 and BHV-4 was highest among cows under 3 years of age [26]. Our findings suggest that this coinfection may be more prevalent in calves under 1 year of age. However, we did not find any correlation between BHV-4 and other pathogens in adult cows, suggesting that coinfection is not a primary factor in the development of reproductive disorders. Among the pathogens responsible for reproductive disorders in dairy cows, the prevalence of BHV-1, BVDV-1, and BVDV-2 was less than 10%. This low prevalence may be attributed to the successful implementation of IBR/BVD immunization programs on these farms. Interestingly, only BHV-4 exhibited a difference in prevalence between calves and cows, suggesting that BHV-4 was correlated with the reproductive status of cows, whereas BHV-1, BVDV-1, and BVDV-2 were not. This finding also suggests that BHV-4 may be the primary pathogen responsible for reproductive disorders.

The association between BHV-4 and reproductive disorders is controversial. Most studies have reported a relatively high detection rate of BHV-4 in dairy cows with reproductive disorders [8, 31–33]. These findings do not directly explain the correlation between BHV-4 and such disorders in dairy cows. In our study, statistical analysis revealed no association between BHV-4 antibodies and reproductive disorders in dairy cows. However, we did observe a direct correlation between the presence of BHV-4 nucleic acid and reproductive disorders in dairy cows, which is consistent with findings from previous research [3, 30]. Given the high prevalence of BHV-4 antibodies and nucleic acid in Chinese dairy cows, prioritizing the prevention and control of BHV-4 is crucial for reducing reproductive disorders and minimizing economic losses.

Nevertheless, research has also suggested that positive BHV-4 antibodies and nucleic acids do not always correlate with reproductive disorders in dairy cows [13, 34]. This discrepancy may be due to regional variations as well as differences in sample types and sources. In our study, a significant number of cows tested positive for both BHV-4 antibodies and nucleic acids but did not display any clinical symptoms. This finding indicates that BHV-4 typically causes subclinical infections and remains latent, potentially leading to reproductive disorders only when the immune system is weakened or exposed to specific stimuli. In the absence of a BHV-4 vaccine, improving the immune status of pregnant cows and minimizing their stress responses may be effective in reducing the occurrence of reproductive disorders. To the best of our knowledge, our study is one of the few that has examined the correlations between BHV-1, BHV-4, BVDV-1, and BVDV-2 and reproductive disorders in dairy cows. The inclusion of these key viral pathogens in the analysis allows for more precise etiological conclusions. However, a limitation of this study is its exclusion of bacterial agents associated with reproductive disorders on the basis of the general perception that BHV-4 acts as a cofactor in bacterial inflammatory responses [35]. Additionally, our study revealed no significant effects of BHV-1, BHV-4, BVDV-1, or BVDV-2 on average calf birth weight or average daily milk yield in dairy cows. This finding contradicts the results of Areda, Chigerwe, and Crossley [3], which may be attributed to the limited data available for analysis in our study.

The vertical transmission of BHV-4 has been reported in previous studies [8, 34]. In this study, BHV-4 DNA was also detected in various tissues of stillborn fetuses from a BHV-4-positive cow, indicating that the infection likely originated from the mother, as the fetus had no exposure to the external environment. Vertical transmission means that infection can be passed from the dam to the fetus, thereby increasing the risk of infection in newborn calves within the herd, necessitating effective management of the health status of dams to prevent the transmission of pathogens to fetuses during gestation. Reducing vertical transmission requires more stringent monitoring and screening of breeding cattle to ensure that only BHV-4-negative animals enter the breeding population. Additionally, vertical transmission suggests that vaccination protocols may need adjustment, with booster vaccinations before breeding and during pregnancy potentially being more effective in preventing vertical transmission.

We also investigated the histotropism of BHV-4 in BHV-4-positive cows and their fetal tissues. Our findings revealed increased levels of BHV-4 in the blood, lymphoid tissue, vulva, vaginal wall, uterine body, and cervix. However, lower levels were observed in the uterine horns, suggesting that BHV-4 primarily colonizes the reproductive tract and lymphocytes in cows. In stillborn fetuses, heightened levels of BHV-4 were observed in both the cervix and uterine body. Histopathological sections confirmed uterine and vaginal mucosal damage in BHV-4-infected stillborn fetuses, a phenomenon not previously reported. The tropism of BHV-4 for blood and the reproductive tract implies that monitoring BHV-4 through both blood samples and reproductive tract swabs can increase the accuracy of detection. Furthermore, understanding the tissue tropism of BHV-4 can aid in the design of more effective drug delivery systems. Specifically, targeted drug delivery systems for the reproductive tract or lymph nodes can be developed to improve treatment efficacy and reduce side effects on healthy tissues.

This study provides important theoretical guidance for the development of BHV-4 vaccines and the formulation of immunization strategies. Understanding the distribution of BHV-4 in specific tissues facilitates the design of targeted vaccines and the development of local administration methods, such as vaginal or intrauterine vaccination. These methods can induce a robust immune response directly at infection sites, effectively preventing infection and transmission in the reproductive tract. Additionally, the study revealed that antibodies induced by live BHV-4 virus have neutralizing effects, suggesting that attenuated or inactivated vaccines could be effective strategies for vaccine development. Given the susceptibility of BHV-4 to infection during the first parity and its potential for vertical transmission, the vaccination of cows before or early in pregnancy could enhance both maternal and fetal immune protection, thereby reducing the risk of BHV-4 transmission within the herd and minimizing vertical transmission.

## 5. Conclusions

Our study revealed a substantial prevalence of BHV-4 across the eight farms surveyed, with its infection intricately linked to the breeding status of cows. Moreover, this study has substantiated the correlation between BHV-4 and reproductive disorders in dairy cows. Additionally, our findings suggest that BHV-4 can induce damage to the uterine and vaginal mucosa of fetal cattle through vertical transmission. In conclusion, our results provide a novel perspective for delving into the interconnection between BHV-4 and reproductive disorders and shedding light on the transmission route of this virus.

## Figures and Tables

**Table 1:** Association between BHV-4 and BHV-1, BVDV-1, and BVDV-2.

	BHV-4 nucleic acid status all herds		BHV-4 nucleic acid status calf herds		BHV-4 nucleic acid status cow herds	
	Negative	Positive	Negative	Positive	Negative	Positive
BHV-1 nucleic acid status	*p*=0.003^a^		*p*=0.001^a^		*p*=0.265	
Negative	235	122	137	16	98	106
Positive	11	18	4	5	7	13
BVDV-1 nucleic acid status	*p*=0.91		*p*=0.209		*p*=0.445	
Negative	224	127	130	17	94	110
Positive	22	13	11	4	11	9
BVDV-2 nucleic acid status	*p*=0.41		*p*=1.000		*p*=0.913	
Negative	240	139	137	21	103	118
Positive	6	1	4	0	2	1

^a^Statistically significant (*p* < 0.05).

**Table 2:** Correlation analysis of BHV-1 and BHV-4 with reproductive disorders.

	BHV-1 nucleic acid status cow herds		BHV-4 nucleic acid status cow herds		BHV-4 antibody status cow herds	
	Negative	Positive	Negative	Positive	Negative	Positive
Reproductive disorders	*p*=0.253		*p*=0.042^a^		*p*=0.830	
Negative	87	2	47	42	12	77
Positive	8	1	1	8	2	7

^a^Statistically significant (*p* < 0.05).

**Table 3:** Correlation analysis of BVDV-1 and BVDV-2 with reproductive disorders.

	BVDV-1 nucleic acid status cow herds		BVDV-2 nucleic acid status cow herds	
	Negative	Positive	Negative	Positive
Reproductive disorders	*p*=0.587		*p*=1.000	
Negative	80	9	87	2
Positive	7	2	9	0

**Table 4:** CT values of BHV-4 in different tissues of BHV-4-positive cows.

Tissues	CT values
Heart	32.913
Liver	35.235
Spleen	32.398
Lung	34.238
Kidney	35.543
Lymphoid tissue	27.779
Blood	21.116
Uterine horns	30.985
Cervix uteri	15.094
Uterine body	15.318
Vulva	18.109
Vaginal wall	22.275

**Table 5:** CT values of BHV-4 in different stillborn fetal tissues.

Tissues	CT values
Heart	35.13
Liver	—
Lung	—
Kidney	36.04
Pancreas	—
Trachea	—
Muscle	—
Pericardium	—
Umbilical cord	28.87
Medulla oblongata	—
Brain	35.57
Rumen	24.9
Abomasum	36.81
Duodenum	33.84
Jejunum	—
Ileum	31.85
Cecum	34.07
Colon	35.02
Breast	—
Vulva	34.41
Cervix uteri	24.06
Uterine body	20.21

## Data Availability

The data that support the findings of this study are available from the corresponding author upon reasonable request.

## Supporting Information

Supporting Information 1 Detailed prevalence data of BHV-1, BHV-4, BVDV-1, and BVDV-2.

Supporting Information 2 Reproductive information.
