# Supplementary material for: Unveiling the Role of Bovine Herpesvirus Type 4 (BHV-4) in Dairy Cow Reproductive Disorders: Insights From a Multifaceted Study in China
Source: Transbound Emerg Dis. 2024 Dec 27;2024:4048149. doi: 10.1155/tbed/4048149 (PMC12016887; doi:10.1155/tbed/4048149)
Supplement: Supporting Information 1 — Detailed prevalence data of BHV-1, BHV-4, BVDV-1, and BVDV-2. [file 4048149.f1.docx]

Table S1. The identification of different pathogens in calves.

|  | Cow number | Sample number | Antibody | Nucleic acid | | | |
| --- | --- | --- | --- | --- | --- | --- | --- |
|  |  |  | BHV-4 | BHV-4 | BHV-1 | BVDV-1 | BVDV-2 |
| Farm A | 243002 | LY-1 | - | - | - | - | - |
|  | 243036 | LY-2 | - | - | - | - | - |
|  | 242990 | LY-3 | - | - | - | - | - |
|  | 243016 | LY-4 | - | - | - | - | - |
|  | 242742 | LY-5 | - | - | - | - | - |
|  | 242858 | LY-6 | - | - | - | - | - |
|  | 243000 | LY-7 | - | - | - | - | - |
|  | 242842 | LY-8 | - | - | - | - | - |
|  | 242888 | LY-9 | - | - | - | - | - |
|  | 242822 | LY-10 | - | - | - | - | - |
|  | 242755 | LY-11 | - | - | - | - | - |
|  | 242751 | LY-12 | - | - | - | - | - |
|  | 230038 | LY-13 | - | - | - | - | - |
|  | 243060 | LY-14 | - | - | - | - | - |
|  | 242070 | LY-15 | - | - | - | - | - |
|  | 242743 | LY-16 | - | - | - | + | - |
|  | 243054 | LY-17 | - | - | - | - | - |
|  | 242870 | LY-18 | - | - | - | - | - |
|  | 242814 | LY-19 | - | - | - | - | - |
|  | 242834 | LY-20 | - | - | - | - | - |
| Farm B | 221179 | JY-1 | - | - | - | - | - |
|  | 221336 | JY-2 | - | - | - | - | - |
|  | 221243 | JY-3 | - | - | - | - | - |
|  | 221172 | JY-4 | + | - | + | + | - |
|  | 221111 | JY-5 | - | - | + | - | - |
|  | 221101 | JY-6 | - | - | + | - | - |
|  | 221141 | JY-7 | - | - | - | - | - |
|  | 221374 | JY-8 | - | - | - | - | - |
|  | 221100 | JY-9 | - | - | - | - | - |
|  | 221181 | JY-10 | - | - | - | - | - |
|  | 221151 | JY-11 | - | - | - | - | - |
|  | 221209 | JY-12 | - | - | - | - | - |
|  | 221316 | JY-13 | - | - | - | - | - |
|  | 221127 | JY-14 | - | - | - | - | - |
|  | 221183 | JY-15 | - | - | - | - | - |
|  | 221230 | JY-16 | - | - | - | + | - |
|  | 221143 | JY-17 | - | + | - | - | - |
|  | 221224 | JY-18 | - | - | - | - | - |
|  | 221190 | JY-19 | - | - | - | + | - |
|  | 221196 | JY-20 | - | - | - | - | - |
| Farm C | 706751 | SQ-24 | + | + | - | - | - |
|  | 806483 | SQ-25 | + | + | - | - | - |
|  | 239646 | SQ-26 | - | - | - | - | - |
|  | 239408 | SQ-27 | - | - | - | - | - |
|  | 239520 | SQ-28 | + | - | - | - | - |
|  | 239469 | SQ-29 | - | - | - | - | - |
|  | 239459 | SQ-30 | - | - | - | - | - |
|  | 239564 | SQ-31 | - | - | - | - | - |
|  | 239523 | SQ-32 | - | - | - | - | - |
|  | 239638 | SQ-33 | - | - | - | + | - |
|  | 239624 | SQ-34 | - | - | - | - | - |
|  | 239614 | SQ-35 | - | - | - | - | - |
|  | 239550 | SQ-36 | - | - | - | - | - |
|  | 239666 | SQ-37 | - | - | - | - | - |
|  | 239962 | SQ-38 | - | - | - | - | - |
|  | 239373 | SQ-39 | - | - | - | + | - |
|  | 239411 | SQ-40 | - | - | - | + | + |
|  | 239532 | SQ-41 | - | - | - | - | - |
|  | 239412 | SQ-42 | - | - | - | - | + |
|  | 239572 | SQ-43 | - | - | - | - | - |
|  | 239512 | SQ-44 | - | - | - | - | - |
|  | 239371 | SQ-45 | - | - | - | - | - |
| Farm D | 222303 | NP-31 | - | - | - | - | - |
|  | 222314 | NP-32 | - | + | + | - | - |
|  | 222327 | NP-33 | - | - | - | - | - |
|  | 222331 | NP-34 | + | - | - | - | - |
|  | 222337 | NP-35 | - | - | - | - | - |
|  | 222342 | NP-36 | - | - | - | - | - |
|  | 222344 | NP-37 | - | - | - | - | - |
|  | 222351 | NP-38 | - | - | - | - | - |
|  | 222360 | NP-39 | - | - | - | - | - |
|  | 222363 | NP-40 | - | - | - | - | - |
|  | 222369 | NP-41 | - | - | - | - | - |
|  | 222371 | NP-42 | - | - | - | - | - |
|  | 223119 | NP-43 | - | - | - | - | - |
|  | 223142 | NP-44 | - | - | - | - | - |
|  | 222372 | NP-45 | - | - | - | - | - |
|  | 22533 | NP-46 | - | - | - | - | - |
|  | 22538 | NP-47 | - | - | - | - | - |
|  | 22554 | NP-48 | - | - | - | - | - |
|  | 22562 | NP-49 | - | - | - | - | - |
|  | 22580 | NP-50 | - | + | - | + | - |
| Farm E | 255234 | QJ-1 | + | - | - | - | - |
|  | 255244 | QJ-2 | + | - | - | - | - |
|  | 255233 | QJ-3 | - | - | - | - | - |
|  | 255239 | QJ-4 | + | - | - | - | - |
|  | 255225 | QJ-5 | - | - | - | - | - |
|  | 255273 | QJ-6 | - | - | - | - | - |
|  | 255269 | QJ-7 | + | - | - | + | - |
|  | 255295 | QJ-8 | - | - | - | - | - |
|  | 255260 | QJ-9 | + | - | - | - | - |
|  | 255226 | QJ-10 | - | - | - | - | - |
|  | 255262 | QJ-11 | + | - | - | + | - |
|  | 255223 | QJ-12 | + | - | - | - | - |
|  | 255221 | QJ-13 | - | - | - | - | - |
|  | 255242 | QJ-14 | + | - | - | - | - |
|  | 255290 | QJ-15 | + | - | - | - | - |
|  | 255217 | QJ-16 | + | - | - | - | - |
|  | 255213 | QJ-17 | - | - | - | - | - |
|  | 255272 | QJ-18 | + | + | - | - | - |
|  | 255210 | QJ-19 | + | - | - | - | - |
|  | 255292 | QJ-20 | - | - | - | - | - |
| Farm F | 151114 | HY-1 | + | + | + | - | - |
|  | 196224 | HY-2 | + | - | - | - | - |
|  | 185302 | HY-3 | + | + | + | + | - |
|  | 209488 | HY-4 | + | + | - | - | - |
|  | 196205 | HY-5 | + | + | - | - | - |
|  | 209950 | HY-6 | + | - | - | - | - |
|  | 223744 | HY-7 | - | - | - | - | - |
|  | 223760 | HY-8 | - | - | - | - | - |
|  | 223755 | HY-9 | - | - | - | - | - |
|  | 223780 | HY-10 | - | - | - | - | - |
|  | 209760 | HY-11 | + | - | - | - | - |
|  | 209591 | HY-12 | - | - | - | - | - |
|  | 223796 | HY-13 | - | - | - | - | - |
|  | 223791 | HY-14 | - | - | - | - | - |
|  | 173495 | HY-15 | + | - | - | - | - |
|  | 196308 | HY-16 | + | + | - | - | - |
|  | 223751 | HY-17 | - | - | - | - | - |
|  | 223772 | HY-18 | - | - | - | - | - |
|  | 223769 | HY-19 | - | - | - | - | - |
|  | 184682 | HY-20 | + | + | - | - | - |
| Farm G | 221961 | ZW-24 | - | + | - | + | - |
|  | 221968 | ZW-25 | - | + | - | - | - |
|  | 221979 | ZW-26 | - | - | - | - | - |
|  | 221992 | ZW-27 | - | - | - | - | - |
|  | 221994 | ZW-28 | - | + | + | - | - |
|  | 221996 | ZW-29 | - | - | - | - | - |
|  | 222001 | ZW-30 | - | - | - | - | - |
|  | 222005 | ZW-31 | - | - | - | - | - |
|  | 222006 | ZW-32 | - | + | + | - | - |
|  | 222011 | ZW-33 | - | - | - | + | - |
|  | 222017 | ZW-34 | - | - | - | - | - |
|  | 222018 | ZW-35 | - | - | - | - | - |
|  | 222020 | ZW-36 | - | - | - | - | - |
|  | 222027 | ZW-37 | - | - | - | - | - |
|  | 222030 | ZW-38 | - | - | - | - | - |
|  | 222034 | ZW-39 | - | - | - | - | - |
|  | 222053 | ZW-40 | - | - | - | - | - |
|  | 226139 | ZW-41 | - | - | - | - | - |
|  | 226269 | ZW-42 | - | - | - | - | - |
|  | 222043 | ZW-43 | - | - | - | - | - |
| Farm H | 226805 | BJ-31 | - | - | - | - | - |
|  | 226887 | BJ-32 | - | - | - | - | - |
|  | 226902 | BJ-33 | - | - | - | - | - |
|  | 226903 | BJ-34 | + | - | - | + | - |
|  | 226923 | BJ-35 | - | - | - | - | - |
|  | 226929 | BJ-36 | - | + | - | + | - |
|  | 226959 | BJ-37 | - | - | + | - | - |
|  | 226973 | BJ-38 | - | + | - | - | - |
|  | 226975 | BJ-39 | - | - | - | - | - |
|  | 226996 | BJ-40 | - | + | - | - | - |
|  | 227001 | BJ-41 | - | - | - | - | - |
|  | 227008 | BJ-42 | - | - | - | - | - |
|  | 227057 | BJ-43 | - | - | - | - | - |
|  | 227092 | BJ-44 | - | - | - | - | + |
|  | 227105 | BJ-45 | - | + | - | - | - |
|  | 227110 | BJ-46 | - | + | - | - | - |
|  | 227139 | BJ-47 | - | - | - | - | - |
|  | 227151 | BJ-48 | - | - | - | - | - |
|  | 227275 | BJ-49 | - | - | - | - | + |
|  | 227297 | BJ-50 | - | - | - | - | - |

Table S2. The identification of different pathogens in cows.

|  | Cow number | Sample number | Antibody | Nucleic acid | | | |
| --- | --- | --- | --- | --- | --- | --- | --- |
|  |  |  | BHV-4 | BHV-4 | BHV-1 | BVDV-1 | BVDV-2 |
| Farm A | 121336 | LY-21 | - | + | - | - | - |
|  | 809660 | LY-22 | + | - | - | - | - |
|  | 401855 | LY-23 | - | - | - | - | - |
|  | 615301 | LY-24 | + | - | - | - | - |
|  | 121156 | LY-25 | + | + | - | - | - |
|  | 121338 | LY-26 | - | + | - | - | - |
|  | 121325 | LY-27 | - | + | - | - | - |
|  | 615196 | LY-28 | + | - | - | - | - |
|  | 908721 | LY-29 | + | - | - | - | - |
|  | 121376 | LY-30 | - | + | - | - | - |
|  | 209800 | LY-31 | + | - | - | - | - |
|  | 121372 | LY-32 | - | - | - | - | - |
|  | 714489 | LY-33 | + | - | - | - | - |
|  | 209793 | LY-34 | + | + | - | - | - |
|  | 909444 | LY-35 | + | - | - | - | - |
|  | 614632 | LY-36 | + | - | - | - | - |
|  | 208562 | LY-37 | + | - | - | - | - |
|  | 121331 | LY-38 | + | + | - | - | - |
|  | 121312 | LY-39 | - | - | - | - | - |
|  | 614857 | LY-40 | + | - | - | - | - |
|  | 121162 | LY-41 | + | + | - | + | - |
|  | 714812 | LY-42 | + | - | - | - | - |
|  | 908564 | LY-43 | - | - | - | - | - |
|  | 908940 | LY-44 | + | - | - | - | - |
|  | 809195 | LY-45 | + | + | - | - | - |
|  | 209820 | LY-46 | + | - | - | - | - |
|  | 208599 | LY-47 | + | + | - | - | - |
|  | 808220 | LY-48 | + | - | - | - | - |
|  | 241403 | LY-49 | - | - | - | - | - |
|  | 208428 | LY-50 | + | + | - | - | - |
| Farm B | 201744 | JY-21 | + | + | + | - | - |
|  | 201740 | JY-22 | + | + | - | - | - |
|  | 201843 | JY-23 | + | + | - | - | - |
|  | 201765 | JY-24 | + | + | + | - | - |
|  | 200617 | JY-25 | + | - | - | - | - |
|  | 200700 | JY-26 | + | + | + | - | - |
|  | 200303 | JY-27 | + | + | - | - | - |
|  | 202165 | JY-28 | + | - | + | - | - |
|  | 201669 | JY-29 | + | + | + | - | - |
|  | 201317 | JY-30 | + | + | - | - | - |
|  | 206042 | JY-31 | - | - | - | - | - |
|  | 201268 | JY-32 | + | - | - | - | - |
|  | 200922 | JY-33 | + | + | - | - | - |
|  | 200107 | JY-34 | + | + | + | - | - |
|  | 202671 | JY-35 | + | + | - | - | - |
|  | 200913 | JY-36 | + | + | - | - | - |
|  | 201813 | JY-37 | + | + | - | - | - |
|  | 200831 | JY-38 | - | - | - | - | - |
|  | 201339 | JY-39 | + | + | + | - | - |
|  | 201908 | JY-40 | + | + | - | - | - |
|  | 201354 | JY-41 | + | + | + | - | - |
|  | 206067 | JY-42 | + | + | + | - | - |
|  | 205375 | JY-43 | + | + | - | - | - |
|  | 201952 | JY-44 | + | + | + | - | - |
|  | 201525 | JY-45 | + | + | + | - | - |
|  | 201916 | JY-46 | + | + | - | - | - |
|  | 200752 | JY-47 | - | - | - | - | - |
| Farm C | 207838 | SQ-1 | + | + | - | + | - |
|  | 906283 | SQ-2 | + | + | - | - | - |
|  | 105744 | SQ-3 | + | - | - | - | - |
|  | 208047 | SQ-4 | + | - | - | - | - |
|  | 806998 | SQ-5 | + | - | - | - | - |
|  | 208226 | SQ-6 | + | - | - | - | - |
|  | 907711 | SQ-7 | + | + | - | + | + |
|  | 707574 | SQ-8 | + | + | - | - | - |
|  | 207903 | SQ-9 | + | + | - | - | - |
|  | 208043 | SQ-10 | + | + | - | - | - |
|  | 208139 | SQ-11 | + | - | - | - | - |
|  | 205963 | SQ-12 | + | + | - | - | - |
|  | 105671 | SQ-13 | + | + | - | - | - |
|  | 906863 | SQ-14 | + | + | - | - | - |
|  | 208209 | SQ-15 | + | - | - | - | - |
|  | 207696 | SQ-16 | + | + | - | - | - |
|  | 707190 | SQ-17 | + | + | - | - | - |
|  | 105760 | SQ-18 | + | + | - | - | - |
|  | 706241 | SQ-19 | + | + | - | - | - |
|  | 208174 | SQ-20 | + | + | - | - | - |
|  | 807004 | SQ-21 | + | - | - | - | - |
|  | 907001 | SQ-22 | + | + | - | - | - |
|  | 706538 | SQ-23 | + | - | - | - | - |
| Farm D | 3181 | NP-1 | + | + | - | - | - |
|  | 5041 | NP-2 | + | + | - | - | - |
|  | 5183 | NP-3 | + | + | - | - | - |
|  | 6166 | NP-4 | + | + | - | - | - |
|  | 7200 | NP-5 | + | - | - | - | - |
|  | 7297 | NP-6 | + | - | - | - | - |
|  | 7318 | NP-7 | + | + | - | - | - |
|  | 8019 | NP-8 | + | + | - | + | - |
|  | 8305 | NP-9 | + | + | - | - | - |
|  | 19012 | NP-10 | + | - | - | - | - |
|  | 19354 | NP-11 | + | + | - | + | - |
|  | 19379 | NP-12 | + | + | - | - | - |
|  | 19516 | NP-13 | + | + | - | - | - |
|  | 19578 | NP-14 | + | + | - | - | - |
|  | 20042 | NP-15 | + | + | + | - | - |
|  | 190034 | NP-16 | + | - | + | - | - |
|  | 190084 | NP-17 | + | + | - | - | - |
|  | 190184 | NP-18 | + | - | - | - | - |
|  | 210153 | NP-19 | + | + | + | - | - |
|  | 210163 | NP-20 | + | - | - | - | - |
|  | 210181 | NP-21 | + | - | - | + | - |
|  | 210182 | NP-22 | + | + | - | - | - |
|  | 210234 | NP-23 | + | + | - | - | - |
|  | 210320 | NP-24 | + | + | - | - | - |
|  | 210345 | NP-25 | + | + | - | - | - |
|  | 210384 | NP-26 | + | - | - | - | - |
|  | 210402 | NP-27 | + | + | - | - | - |
|  | 210429 | NP-28 | + | - | - | - | - |
|  | 210433 | NP-29 | + | - | + | - | - |
|  | 210445 | NP-30 | + | + | - | - | - |
| Farm E | 206333 | QJ-21 | + | - | - | - | - |
|  | 205366 | QJ-22 | + | + | - | - | - |
|  | 161490 | QJ-23 | + | - | - | - | - |
|  | 207578 | QJ-24 | + | + | - | - | - |
|  | 205084 | QJ-25 | + | - | - | - | - |
|  | 206394 | QJ-26 | + | - | - | - | - |
|  | 907220 | QJ-27 | + | - | - | - | - |
|  | 210176 | QJ-28 | + | - | + | - | - |
|  | 207428 | QJ-29 | + | - | - | + | - |
|  | 206091 | QJ-30 | - | - | - | - | - |
|  | 907462 | QJ-31 | + | - | - | - | - |
|  | 206708 | QJ-32 | + | + | - | - | - |
|  | 206525 | QJ-33 | + | - | - | - | - |
|  | 206549 | QJ-34 | + | + | - | - | - |
|  | 205026 | QJ-35 | + | + | - | - | - |
|  | 161468 | QJ-36 | + | - | - | - | - |
|  | 210233 | QJ-37 | + | - | - | - | - |
|  | 907672 | QJ-38 | + | - | - | - | - |
|  | 210060 | QJ-39 | - | - | - | - | - |
|  | 210258 | QJ-40 | + | + | - | - | - |
|  | 907612 | QJ-41 | + | + | - | - | - |
|  | 206405 | QJ-42 | + | - | - | - | - |
|  | 206485 | QJ-43 | + | - | - | - | - |
|  | 907384 | QJ-44 | + | + | - | + | - |
|  | 161470 | QJ-45 | + | - | - | - | - |
|  | 210360 | QJ-46 | - | - | + | - | - |
|  | 206055 | QJ-47 | + | - | - | - | - |
|  | 210170 | QJ-48 | - | - | - | + | - |
|  | 206174 | QJ-49 | + | + | - | - | - |
|  | 210128 | QJ-50 | + | + | - | - | - |
| Farm F | 196010 | HY-21 | + | + | - | - | - |
|  | 172359 | HY-22 | + | - | - | - | - |
|  | 223781 | HY-23 | - | - | - | - | - |
|  | 161176 | HY-24 | + | + | - | - | - |
|  | 184905 | HY-25 | + | - | - | - | - |
|  | 223765 | HY-26 | - | - | - | - | - |
|  | 185058 | HY-27 | + | - | - | - | - |
|  | 223749 | HY-28 | - | - | - | - | - |
|  | 172330 | HY-29 | + | - | - | - | - |
|  | 223809 | HY-30 | - | - | - | - | - |
|  | 209811 | HY-31 | + | - | - | - | - |
|  | 223775 | HY-32 | - | - | - | + | - |
|  | 223776 | HY-33 | - | - | - | - | - |
|  | 210125 | HY-34 | - | + | - | - | - |
|  | 210006 | HY-35 | + | + | - | - | - |
|  | 197374 | HY-36 | + | - | - | + | - |
|  | 185585 | HY-37 | + | - | - | - | - |
|  | 209306 | HY-38 | + | + | - | - | - |
|  | 185633 | HY-39 | + | + | - | - | - |
|  | 196454 | HY-40 | + | + | - | - | - |
|  | 208049 | HY-41 | - | + | - | - | - |
|  | 223802 | HY-42 | - | - | - | - | - |
|  | 209874 | HY-43 | - | + | - | - | - |
|  | 185136 | HY-44 | + | + | - | - | - |
|  | 184689 | HY-45 | - | - | - | - | - |
|  | 223757 | HY-46 | + | + | - | - | - |
|  | 172896 | HY-47 | + | + | - | - | - |
|  | 223798 | HY-48 | + | - | - | - | - |
|  | 223748 | HY-49 | - | - | - | - | - |
|  | 223746 | HY-50 | + | + | - | - | - |
| Farm G | 68013 | ZW-1 | + | - | - | - | - |
|  | 68014 | ZW-2 | + | - | - | - | - |
|  | 170137 | ZW-3 | + | + | - | - | - |
|  | 170626 | ZW-4 | - | + | - | + | - |
|  | 180601 | ZW-5 | + | + | - | - | - |
|  | 180772 | ZW-6 | + | - | - | - | - |
|  | 180851 | ZW-7 | + | - | - | - | - |
|  | 181160 | ZW-8 | + | - | - | - | - |
|  | 190134 | ZW-9 | + | + | - | - | - |
|  | 190296 | ZW-10 | + | + | - | - | - |
|  | 190460 | ZW-11 | + | - | - | - | - |
|  | 190605 | ZW-12 | + | + | - | - | - |
|  | 190626 | ZW-13 | + | + | + | - | - |
|  | 190769 | ZW-14 | + | + | - | - | - |
|  | 190845 | ZW-15 | + | + | - | - | - |
|  | 191091 | ZW-16 | + | + | - | - | - |
|  | 191125 | ZW-17 | + | - | - | - | - |
|  | 200038 | ZW-18 | + | - | - | - | - |
|  | 201033 | ZW-19 | + | - | - | - | - |
|  | 201367 | ZW-20 | + | - | - | - | - |
|  | 201376 | ZW-21 | + | + | - | - | - |
|  | 201393 | ZW-22 | + | - | - | + | - |
|  | 210088 | ZW-23 | + | + | - | + | - |
|  | 190301 | ZW-44 | + | - | - | - | - |
| Farm H | 160551 | BJ-1 | + | - | - | + | - |
|  | 161036 | BJ-2 | + | + | - | - | - |
|  | 162900 | BJ-3 | + | - | - | + | - |
|  | 170810 | BJ-4 | + | - | - | - | - |
|  | 173096 | BJ-5 | + | - | - | - | - |
|  | 174404 | BJ-6 | + | - | - | - | + |
|  | 180513 | BJ-7 | + | - | + | + | - |
|  | 180734 | BJ-8 | + | - | - | - | - |
|  | 181793 | BJ-9 | + | + | - | - | - |
|  | 181903 | BJ-10 | + | + | - | - | - |
|  | 182047 | BJ-11 | + | + | - | - | - |
|  | 190180 | BJ-12 | + | + | - | + | - |
|  | 190361 | BJ-13 | + | - | - | + | + |
|  | 190574 | BJ-14 | + | + | - | - | - |
|  | 191215 | BJ-15 | + | - | - | + | - |
|  | 191685 | BJ-16 | + | + | - | - | - |
|  | 192294 | BJ-17 | + | + | - | - | - |
|  | 192969 | BJ-18 | + | + | - | - | - |
|  | 193019 | BJ-19 | + | - | - | - | - |
|  | 193176 | BJ-20 | + | - | - | - | - |
|  | 193338 | BJ-21 | + | - | - | - | - |
|  | 200083 | BJ-22 | + | + | - | - | - |
|  | 200681 | BJ-23 | + | + | - | - | - |
|  | 200710 | BJ-24 | + | - | + | - | - |
|  | 204238 | BJ-25 | + | + | - | - | - |
|  | 205460 | BJ-26 | + | + | - | - | - |
|  | 210246 | BJ-27 | + | + | - | - | - |
|  | 210363 | BJ-28 | + | + | - | - | - |
|  | 210364 | BJ-29 | + | + | - | - | - |
|  | 210570 | BJ-30 | + | + | - | - | - |

| Farm | BHV-4 antibody-positive rate | BHV-4 antibody-positive rate in cows | BHV-4 antibody-positive rate in calves |
| --- | --- | --- | --- |
| Farm A | 21/50(42.0%) | 21/30(70.0%) | 0/20(0.0%) |
| Farm B | 25/47(53.2%) | 24/27(88.9%) | 1/20(5.0%) |
| Farm C | 26/45(57.8%) | 23/23(100%) | 3/22(13.6%) |
| Farm D | 31/50（62.0%） | 30/30（100%） | 1/20(5.0%) |
| Farm E | 38/50(76.0%) | 26/30(86.7%) | 12/20(60.0%) |
| Farm F | 28/50(56.0%) | 18/30(60.0%) | 10/20(50.0%) |
| Farm G | 23/44（52.3%） | 23/24（95.8%） | 0/20（0.0%） |
| Farm H | 31/50（62.0%） | 30/30（100%） | 1/20（5.0%） |
| Total | 223/386(57.8%) | 195/224(87.1%) | 28/162(17.3%) |

Table S3. The positive rate of BHV-4 antibody.

Table S4. The positive rate of BHV-4 nucleic acid.

| Farm | BHV-4 nucleic acid-positive rate | BHV-4 nucleic acid-positive rate in cows | BHV-4 nucleic acid-positive rate in calves |
| --- | --- | --- | --- |
| Farm A | 11/50(22.0%) | 11/30(36.7%) | 0/20(0.0%) |
| Farm B | 22/47(46.8%) | 21/27(77.8%) | 1/20(5.0%) |
| Farm C | 17/45（37.8%） | 15/23（65.2%） | 2/22（9.1%) |
| Farm D | 22/50（44.0%） | 20/30(66.7%) | 2/20(10.0%) |
| Farm E | 11/50(22.0%) | 10/30(33.3%) | 1/20(5.0%) |
| Farm F | 19/50(38.0%) | 13/30(43.3%) | 6/20(30.0%) |
| Farm G | 16/44（36.4%） | 12/24（50.0%） | 4/20（20.0%） |
| Farm H | 22/50（44.0%） | 17/30（56.7%） | 5/20（25.0%） |
| Total | 140/386(36.3%) | 119/224(53.1%) | 21/162(13.0%) |

Table S5. The positive rate of BHV-1 nucleic acid.

| Farm | BHV-1 nucleic acid-positive rate | BHV-1 nucleic acid-positive rate in cows | BHV-1 nucleic acid-positive rate in calves |
| --- | --- | --- | --- |
| Farm A | 0/50(0.0%) | 0/30(0.0%) | 0/20(0.0%) |
| Farm B | 14/47(29.8%) | 11/27(40.7%) | 3/20(15.0%) |
| Farm C | 0/45（0%） | 0/23（0%） | 0/22（0%） |
| Farm D | 5/50(10.0%) | 4/30(13.3%) | 1/20(5.0%) |
| Farm E | 2/50(4.0%) | 2/30(6.7%) | 0/20(0.0%) |
| Farm F | 2/50(4.0%) | 0/30(0.0%) | 2/20(10%) |
| Farm G | 3/44（6.8%） | 1/24（4.2%） | 2/20(10%) |
| Farm H | 3/50(6.0% | 2/30(6.7%) | 1/20(5.0%) |
| Total | 29/386(7.5%) | 20/224(8.9%) | 9/162(5.6%) |

Table S6. The positive rate of BVDV-1 nucleic acid.

| Farm | BVDV-1 nucleic acid-positive rate | BVDV-1 nucleic acid-positive rate in cows | BVDV-1 nucleic acid-positive rate in calves |
| --- | --- | --- | --- |
| Farm A | 2/50(4.0%) | 1/30(3.3%) | 1/20(5.0%) |
| Farm B | 3/47(6.4%) | 0/27(0%) | 3/20(15%) |
| Farm C | 5/45（11.1%） | 2/23（8.7%） | 3/22（13.6%） |
| Farm D | 4/50(8.0%) | 3/30(10.0%) | 1/20(5.0%) |
| Farm E | 5/50(10.0%) | 3/30(10.0%) | 2/20(10.0%) |
| Farm F | 3/50(6%) | 2/30(6.7%) | 1/20(5.0%) |
| Farm G | 5/44(11.4%) | 3/24(12.5%) | 2/20(10.0%) |
| Farm H | 8/50(16.0%) | 6/30(20.0%) | 2/20(10.0%) |
| Total | 35/386(9.1%) | 20/224(8.9%) | 15/162(9.3%) |

Table S7. The positive rate of BVDV-2 nucleic acid.

| Farm | BVDV-2 nucleic acid-positive rate | BVDV-2 nucleic acid-positive rate in cows | BVDV-2 nucleic acid-positive rate in calves |
| --- | --- | --- | --- |
| Farm A | 0/50(0.0%) | 0/30(0.0%) | 0/20(0.0%) |
| Farm B | 0/47(0%) | 0/27(0%) | 0/20(0.0%) |
| Farm C | 3/45（6.7%） | 1/23（4.3%) | 2/22（9.1%） |
| Farm D | 0/50(0.0%) | 0/30(0.0%) | 0/20(0.0%) |
| Farm E | 0/50(0.0%) | 0/30(0.0%) | 0/20(0.0%) |
| Farm F | 0/50(0.0%) | 0/30(0.0%) | 0/20(0.0%) |
| Farm G | 0/44(0.0%) | 0/24(0.0%) | 0/20(0.0%) |
| Farm H | 4/50(8.0%) | 2/30(6.7%) | 2/20(10.0%) |
| Total | 7/386(1.8%) | 3/224(1.3%) | 4/162(2.5%) |
