# Supplementary material for: Unveiling the Role of Bovine Herpesvirus Type 4 (BHV-4) in Dairy Cow Reproductive Disorders: Insights From a Multifaceted Study in China
Source: Transbound Emerg Dis. 2024 Dec 27;2024:4048149. doi: 10.1155/tbed/4048149 (PMC12016887; doi:10.1155/tbed/4048149)
Supplement: Supporting Information 2 — Reproductive information. [file 4048149.f2.docx]

Table S8. Reproductive information of 98 cows.

| Farm | Cow number | BHV-4 antibody | BHV-4 nucleic acid | BHV-1 nucleic acid | BVDV-1 nucleic acid | BVDV-2 nucleic acid | Parity | Age in months | Average calf birth weight (kg) | Average daily milk yield (kg) | Postpartum disease |
| --- | --- | --- | --- | --- | --- | --- | --- | --- | --- | --- | --- |
| Farm A | 241403 | - | - | - | - | - | 1 | 23.95 | 38 | No data | - |
| Farm A | 121376 | - | + | - | - | - | 1 | 24.18 | 42 | 19.16129032 | - |
| Farm A | 121372 | - | - | - | - | - | 1 | 24.21 | 30 | No data | - |
| Farm A | 121338 | - | + | - | - | - | 1 | 24.44 | 38 | No data | - |
| Farm A | 121336 | - | + | - | - | - | 1 | 24.47 | 42 | 24.27272727 | Retained placenta |
| Farm A | 121331 | + | + | - | - | - | 1 | 24.51 | 39 | No data | - |
| Farm A | 121325 | - | + | - | - | - | 1 | 24.57 | 37 | 27.53125 | - |
| Farm A | 121312 | - | - | - | - | - | 1 | 24.7 | 36 | 29.78125 | - |
| Farm A | 121162 | + | + | - | + | - | 1 | 25.72 | 35 | 27.57575758 | - |
| Farm A | 121156 | + | + | - | - | - | 1 | 25.79 | 36 | No data | - |
| Farm A | 209820 | + | - | - | - | - | 2 | 36.05 | 44 | No data | - |
| Farm A | 209793 | + | + | - | - | - | 2 | 36.35 | 48 | No data | - |
| Farm A | 208599 | + | + | - | - | - | 3 | 46.25 | 42 | 42.25806452 | - |
| Farm A | 208562 | + | - | - | - | - | 3 | 46.64 | 42 | No data | Left displacement of the abomasum (LDA) |
| Farm A | 208428 | + | + | - | - | - | 3 | 47.8 | 41 | 48.19354839 | - |
| Farm A | 909444 | + | - | - | - | - | 3 | 49.67 | 37 | No data | - |
| Farm A | 908940 | + | - | - | - | - | 3 | 52.47 | 43 | No data | - |
| Farm A | 908721 | + | - | - | - | - | 3 | 54.01 | 44 | No data | - |
| Farm A | 908564 | - | - | - | - | - | 4 | 57.53 | 40 | No data | - |
| Farm A | 809660 | + | - | - | - | - | 4 | 60.23 | 44 | 43.3125 | - |
| Farm A | 809195 | + | + | - | - | - | 4 | 62.66 | 43 | No data | - |
| Farm A | 808220 | + | - | - | - | - | 5 | 71.94 | 39 | 39.34375 | Postpartum paralysis |
| Farm A | 714812 | + | - | - | - | - | 5 | 75.53 | 40 | No data | - |
| Farm A | 714489 | + | - | - | - | - | 6 | 82.01 | 43 | No data | Breast disease |
| Farm A | 615301 | + | - | - | - | - | 6 | 86.68 | 40 | No data | - |
| Farm A | 615196 | + | - | - | - | - | 6 | 87.04 | 36 | No data | - |
| Farm A | 614857 | + | - | - | - | - | 6 | 92.63 | 42 | No data | - |
| Farm A | 614632 | + | - | - | - | - | 7 | 94.11 | 40 | No data | - |
| Farm A | 401855 | - | - | - | - | - | 8 | 111.74 | 40 | No data | - |
| Farm F | 210125 | - | + | - | - | - | 2 | 35.07 | 37.0 | 34.19444444 | - |
| Farm F | 210006 | + | + | - | - | - | 2 | 35.92 | 46.5 | 36 | - |
| Farm F | 209874 | - | + | - | - | - | 2 | 36.48 | 40.5 | 34.97142857 | - |
| Farm F | 209811 | + | - | - | - | - | 2 | 37.07 | 35.5 | 32.82857143 | - |
| Farm F | 209306 | + | + | - | - | - | 2 | 39.7 | 48.5 | 38 | - |
| Farm F | 208049 | - | + | - | - | - | 3 | 47.53 | 42.0 | 48.16216216 | - |
| Farm F | 197374 | + | - | - | + | - | 3 | 50.36 | 38.0 | 41.76470588 | - |
| Farm F | 196454 | + | + | - | - | - | 3 | 55.56 | 37.5 | 32.62857143 | - |
| Farm F | 196010 | + | + | - | - | - | 4 | 59.97 | 38.5 | 41.84210526 | - |
| Farm F | 185633 | + | + | - | - | - | 4 | 60.72 | 45.5 | 38.52777778 | - |
| Farm F | 185585 | + | - | - | - | - | 4 | 61.15 | 42 | 35.71052632 | - |
| Farm F | 185136 | + | + | - | - | - | 3 | 63.62 | 46.5 | 30.97142857 | - |
| Farm F | 185058 | + | - | - | - | - | 4 | 63.91 | 44.0 | 39.79411765 | - |
| Farm F | 184905 | + | - | - | - | - | 4 | 64.57 | 41.5 | 34.85714286 | - |
| Farm F | 184689 | - | - | - | - | - | 4 | 65.66 | 36.0 | 38.21052632 | - |
| Farm F | 172896 | + | + | - | - | - | 5 | 76.15 | 43 | 32.97297297 | - |
| Farm F | 172359 | + | - | - | - | - | 5 | 80.49 | 44.5 | 35.71428571 | - |
| Farm F | 172330 | + | - | - | - | - | 6 | 80.82 | 49.5 | 27.29411765 | - |
| Farm F | 161176 | + | + | - | - | - | 6 | 85.46 | 42.0 | 43.47368421 | Metritis |
| Farm G | 210088 | + | + | - | + | - | 2 | 34.7 | 40.0 | 33.11111111 | - |
| Farm G | 201393 | + | - | - | + | - | 2 | 37.7 | 47.5 | 12.5 | Metritis |
| Farm G | 201376 | + | + | - | - | - | 2 | 37.86 | 45 | 49.86486486 | - |
| Farm G | 201367 | + | - | - | - | - | 2 | 37.93 | 38.5 | 23.87878788 | - |
| Farm G | 201033 | + | - | - | - | - | 2 | 39.97 | 43.0 | 30.74285714 | - |
| Farm G | 200038 | + | - | - | - | - | 3 | 47.27 | 40.0 | 22.77777778 | - |
| Farm G | 191125 | + | - | - | - | - | 3 | 48.16 | 46.0 | 23.15625 | - |
| Farm G | 191091 | + | + | - | - | - | 3 | 48.49 | 42.0 | 38.24324324 | - |
| Farm G | 190845 | + | + | - | - | - | 3 | 51.32 | 35.0 | 42.2972973 | - |
| Farm G | 190769 | + | + | - | - | - | 3 | 51.64 | 44.5 | 33.08333333 | Birth canal strain |
| Farm G | 190626 | + | + | + | - | - | 3 | 52.17 | 39.0 | 13.2 | Metritis |
| Farm G | 190605 | + | + | - | - | - | 3 | 52.24 | 47.5 | 35.86111111 | - |
| Farm G | 190460 | + | - | - | - | - | 3 | 52.83 | 37.5 | 36.09677419 | - |
| Farm G | 190296 | + | + | - | - | - | 3 | 53.62 | 42.0 | 32.80555556 | - |
| Farm G | 190134 | + | + | - | - | - | 3 | 54.7 | 46.5 | 34.09090909 | - |
| Farm G | 180772 | + | - | - | - | - | 4 | 64.01 | 45.0 | 31.91428571 | - |
| Farm G | 180601 | + | + | - | - | - | 4 | 64.57 | 46.5 | 35.64516129 | Metritis |
| Farm G | 170626 | - | + | - | + | - | 5 | 73.26 | 43.0 | 36.81081081 | Retained placenta |
| Farm G | 68014 | + | - | - | - | - | 6 | 89.77 | 36.0 | 44.09090909 | - |
| Farm G | 68013 | + | - | - | - | - | 6 | 89.8 | 44.5 | 47.61290323 | - |
| Farm H | 210570 | + | + | - | - | - | 2 | 34.21 | 40.0 | 27.96875 | - |
| Farm H | 210364 | + | + | - | - | - | 2 | 34.9 | 38.0 | 31.9375 | - |
| Farm H | 210363 | + | + | - | - | - | 2 | 34.9 | 42.0 | 36.16129032 | - |
| Farm H | 210246 | + | + | - | - | - | 2 | 35.26 | 30.5 | 43.31578947 | - |
| Farm H | 205460 | + | + | - | - | - | 2 | 37.07 | 36.0 | 29.76470588 | - |
| Farm H | 204238 | + | + | - | - | - | 2 | 39.47 | 34.5 | 33.81818182 | Retained placenta |
| Farm H | 200710 | + | - | + | - | - | 3 | 46.09 | 45.0 | 42.37142857 | - |
| Farm H | 200681 | + | + | - | - | - | 3 | 46.18 | 49.0 | 46.91176471 | - |
| Farm H | 200083 | + | + | - | - | - | 3 | 47.7 | 34.0 | 10.95121951 | Metritis |
| Farm H | 193338 | + | - | - | - | - | 3 | 51.71 | 23.0 | 10.76190476 | Pneumonia |
| Farm H | 193176 | + | - | - | - | - | 3 | 52.01 | 34.0 | 47.48717949 | - |
| Farm H | 193019 | + | - | - | - | - | 3 | 52.3 | 42.0 | 40.21875 | - |
| Farm H | 192969 | + | + | - | - | - | 3 | 52.37 | 41.5 | 36.75 | - |
| Farm H | 192294 | + | + | - | - | - | 3 | 53.62 | 46.5 | 45.97142857 | - |
| Farm H | 191685 | + | + | - | - | - | 3 | 54.7 | 41.5 | 43.025 | - |
| Farm H | 191215 | + | - | - | + | - | 4 | 56.15 | 32.5 | 39.39393939 | - |
| Farm H | 190574 | + | + | - | - | - | 4 | 58.49 | 57.0 | 43.6 | - |
| Farm H | 190361 | + | - | - | + | + | 4 | 59.14 | 49.0 | 43.47619048 | - |
| Farm H | 190180 | + | + | - | + | - | 4 | 59.61 | 37.0 | 13.9 | - |
| Farm H | 182047 | + | + | - | - | - | 4 | 65.66 | 40.0 | 42.87179487 | - |
| Farm H | 181903 | + | + | - | - | - | 4 | 65.92 | 39.0 | 34.6875 | - |
| Farm H | 181793 | + | + | - | - | - | 4 | 66.12 | 44.5 | 41.78378378 | - |
| Farm H | 180734 | + | - | - | - | - | 5 | 69.31 | 43.0 | 48.16129032 | - |
| Farm H | 180513 | + | - | + | + | - | 5 | 70.1 | 39.0 | 30.35294118 | - |
| Farm H | 174404 | + | - | - | - | + | 5 | 73.22 | 38.5 | 27.66666667 | - |
| Farm H | 173096 | + | - | - | - | - | 5 | 76.38 | 43.5 | 36.29411765 | - |
| Farm H | 170810 | + | - | - | - | - | 5 | 82.07 | 44.0 | 44.75675676 | - |
| Farm H | 162900 | + | - | - | + | - | 6 | 88.03 | 41.0 | 40.38235294 | - |
| Farm H | 161036 | + | + | - | - | - | 6 | 92.14 | 41.5 | 34.19354839 | Hoof disease |
| Farm H | 160551 | + | - | - | + | - | 6 | 94.21 | 47.0 | 36.19444444 | - |

Table S9. Relationship between BHV-4 positive and parity.

| Parity | Number | BHV-4 antibody-positive rate | BHV-4 nucleic acid-positive rate |
| --- | --- | --- | --- |
| 0 parity | N=162 | 17.30% | 13.00% |
| 1 parity | N=10 | 30.00% | 70.00% |
| 2 parity | N=18 | 88.90% | 72.20% |
| 3 parity | N=29 | 96.60% | 58.60% |
| 4 parity | N=18 | 88.90% | 50.00% |
| 5 parity | N=10 | 90.00% | 20.00% |
| 6 parity | N=11 | 100.00% | 18.20% |
